# Synthesis of some novel 4-arylidene pyrazoles as potential antimicrobial agents

**DOI:** 10.1186/2191-2858-3-9

**Published:** 2013-08-28

**Authors:** Poonam Khloya, Pawan Kumar, Arpana Mittal, Neeraj K Aggarwal, Pawan K Sharma

**Affiliations:** 1 Department of Chemistry, Kurukshetra University, Kurukshetra 136119, India; 2 Department of Microbiology, Kurukshetra University, Kurukshetra 136119, India

**Keywords:** Pyrazole, Pyrazolone, Antibacterial activity, Antifungal activity, Sulfonamide

## Abstract

**Background:**

Pyrazole and pyrazolone motifs are well known for their wide range of biological activities such as antimicrobial, anti-inflammatory, and antitumor activities. The incorporation of more than one pharmacophore in a single scaffold is a well known approach for the development of more potent drugs. In the present investigation, a series of differently substituted 4-arylidene pyrazole derivatives bearing pyrazole and pyrazolone pharmacophores in a single scaffold was synthesized.

**Results:**

The synthesis of novel 4-arylidene pyrazole compounds is achieved through Knovenagel condensation between 1,3-diaryl-4-formylpyrazoles and 3-methyl-1-phenyl-1*H*-pyrazol-5-(4*H*)-ones in good yields. All compounds were evaluated for their *in vitro* antimicrobial activity.

**Conclusions:**

A series of 4-arylidene pyrazole derivatives was evaluated for their *in vitro* antimicrobial activity against two Gram-positive (*Bacillus subtilis* and *Staphylococcus aureus*) and two Gram-negative bacteria (*Pseudomonas fluorescens* and *Escherichia coli*), as well as two pathogenic fungal strains (*Candida albicans* and *Saccharomyces cerevisiae*). The majority of the compounds displayed excellent antimicrobial profile against the Gram-positive (*B. subtilis* and *S. aureus*), and some of them are even more potent than the reference drug ciprofloxacin.

## Background

**Scheme 1:**
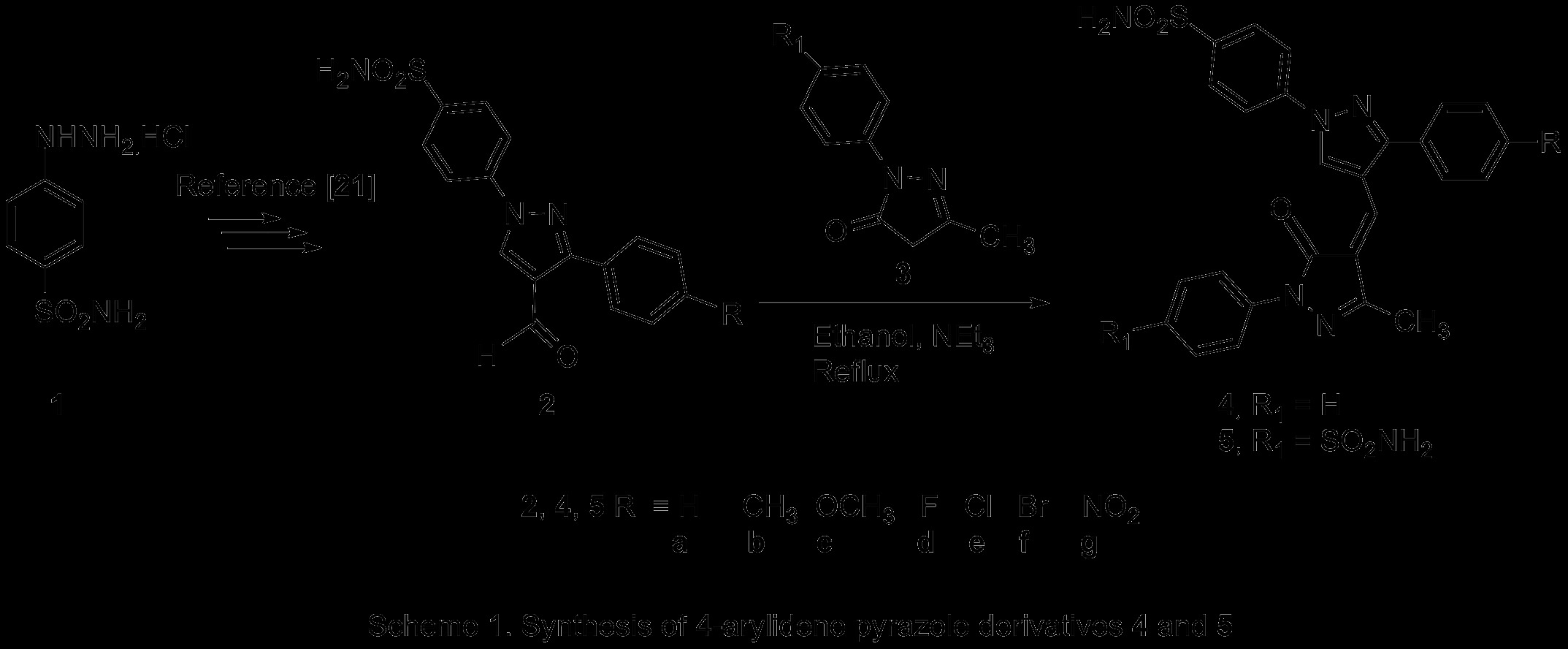
Synthesis of 4-arylidene pyrazole derivatives 4 and 5.

Over the years, excessive use of antimicrobial drugs has led to a worldwide phenomenon of antibacterial resistance. This has resulted into an increase in morbidity and mortality, and has become a worldwide health issue. As a consequence, the development of new antimicrobial agents is in constant demand. The compounds bearing pyrazole nucleus are well known to exhibit versatile range of biological activities such as antimicrobial [1-4], anti-inflammatory [5-7], antidepressant [8], antiviral [9], and antitumor activities [10]. Among these, 4-functionalized pyrazoles occupy a unique position in medicinal chemistry because of their association with antimicrobial [11], anti-inflammatory [12], antiparasitic [13], and antitumor activities [14]. Pyrazol-5-(4*H*)-one also constitutes the core scaffold of various biologically active synthetic heterocyclic compounds which have been associated with some interesting pharmaceutical properties, including analgesic [15], antimicrobial [16,17], anti-inflammatory [18], antitumor [19], and cytotoxicity [20] properties. Understanding that the incorporation of both pyrazole and pyrazolone together in the same scaffold could provide novel compounds with interesting biological activities coupled with our continuing research interest in the field of 4-functionalized pyrazole derivatives [12,21-23] and other biologically active synthetic heterocyclic compounds [24-27], we set out to undertake the synthesis of some novel 4-arylidene pyrazole derivatives bearing benzenesulfonamide moiety at the N1-position of the pyrazole ring (Scheme 1) as potential antimicrobial agents.

## Methods

### *In vitro* antibacterial activity

The agar well diffusion method [28] was used for the determination of antimicrobial activity of all the synthesized compounds. Overnight broth culture of the respective bacterial strains was adjusted to approximately 10^8^ colony forming units (CFU/mL) with sterile distilled water, and 100 μL of diluted inoculum was spread over the petriplates containing 25 mL of nutrient agar media. Eight wells (8 mm in diameter) were made equidistant with each of the plates using a sterile cork borer. The test compounds were dissolved in dimethylsulfoxide (DMSO) and then the antimicrobial effect of the synthesized compounds was evaluated. The wells were filled with 100 μL of the test compound having a concentration of 4.0 mg/mL. The plates were incubated at 37°C for 48 h. The antimicrobial activity was evaluated by measuring the zone of growth inhibition of bacteria surrounding the wells after 24 and 48 h. Ciprofloxacin (4.0 mg/mL) served as the antibacterial control. DMSO was taken as the negative control which did not produce any significant zone of inhibition.

### Determination of minimum inhibitory concentration

The minimum inhibitory concentration (MIC) against the tested bacteria was determined using the macrodilution tube method [29] as recommended by NCCLS (2000). The MIC is the lowest concentration of an antimicrobial compound, which will inhibit the visible growth of a microorganism after an overnight incubation. The MIC of each compound giving an inhibitory zone at a concentration of 4 mg/mL was also tested with the agar well diffusion method. Different concentrations (4,000 to 0.004 μg/mL) of a single compound were applied to the number of wells in the agar plates. The determinations were performed in triplicates, and the results were averaged.

### *In vitro* antifungal activity

The agar well diffusion method was used for the determination of antimicrobial activity of the compounds. Overnight broth culture of the respective fungal strains was adjusted to approximately 10^8^ CFU/mL with sterile distilled water, and 100 μL of diluted inoculum was spread over the petriplates containing 25 mL of Sabouraud's dextrose agar media (pH 5.6). Eight wells (8 mm in diameter) were made equidistant with each of the plates using a sterile cork borer. The test compounds were dissolved in DMSO and then the antimicrobial effect of the test compounds was tested. The wells were filled with 100 μL of the test compound having a concentration 4 mg/mL. The plates were incubated at 30°C for 48 to 72 h. The antimicrobial activity was evaluated by measuring the zone of growth inhibition of fungi surrounding the wells after 48 and 72 h. Fluconazole (4 mg/mL) served as the antifungal control. DMSO was taken as the negative control which did not produce any significant zone of inhibition. The experiments were performed in triplicates. The diameter of the fungal colonies was measured.

## Results and discussion

### Chemistry

The synthetic route used to synthesize the target 4-arylidene pyrazole derivatives (**4** and **5**) is outlined in Scheme 1. 4-Formyl pyrazoles (**2**) were synthesized using our earlier reported procedure [21], while 3-methyl-1-aryl-1*H*-pyrazol-5-(4*H*)-ones (**3**) was prepared by condensation of ethylacetoacetate with appropriate hydrazine [30,31]. Finally, base-catalyzed Knoevenagel condensation of appropriately substituted pyrazol-5-(4*H*)-ones (**3**) with various substituted 4-formylpyrazoles (**2**) in ethanol containing catalytic amount of triethylamine afforded the target 4-arylidene pyrazole derivatives (**4** and **5**) in good yield. Spectral data (IR, ^1^H NMR, and mass) of the newly synthesized compounds **4a-g** and **5a-g** were in full agreement with the proposed structures. In the ^1^H NMR spectra of **4** and **5**, the C=CH proton displayed more downfield signal in the range *δ* 10.18 to 10.25. Besides this, C_5_-H of the pyrazole ring resonates at around *δ* 7.51 to 7.63. The IR spectra of **4** and **5** showed a characteristic absorption band around 1,674 to 1,682 cm^-1^ that was assigned to the C=O stretching, while the two absorptions bands around 1,304 to 1,335 and 1,149 to 1,165 cm^-1^ which further supported the proposed structures of newly synthesized compounds displayed the SO_2_ stretchings.

### Biological evaluation

#### ***In vitro antibacterial activity***

All the synthesized compounds (**4** and **5**) were screened for their *in vitro* antibacterial activity against the four pathogenic bacteria, *Bacillus subtilis* (microbial-type culture collection (MTCC) 121) and *Staphylococcus aureus* (MTCC 96) representing the Gram-positive bacteria, and *Pseudomonas fluorescens* (MTCC 1749) and *Escherichia coli* (MTCC 1652) representing the Gram-negative bacteria (Table 1), by agar well diffusion method [28] using ciprofloxacin as the reference drug. The MIC measurements were performed using a macrodilution method [29] (Table 1).

**Table 1:** ***In vitro *antibacterial activity and MIC of compounds 4 and 5 using agar well diffusion method**

**Compound**^**a**^	**Diameter of zone of inhibition in mm (MIC)**^**b**^			
	***B. subtilis***	***S. aureus***	***P. fluorescens***	***E. coli***
**4a**	28 ± 0.00 (0.04)	26 ± 0.30 (0.4)	14 ± 0.08 (400)	14 ± 0.46 (400)
**4b**	20 ± 0.19 (4.0)	24 ± 0.50 (0.4)	15 ± 0.09 (400)	14 ± 0.23 (400)
**4c**	16 ± 0.19 (40)	24 ± 0.32 (0.4)	-	14 ± 0.23 (400)
**4d**	16 ± 0.00 (40)	12 ± 0.19 (400)	-	14 ± 0.43 (400)
**4e**	12 ± 0.40 (400)	16 ± 0.22 (40)	-	20 ± 0.30 (4)
**4f**	24 ± 0.07 (0.4)	20 ± 0.37 (4.0)	14 ± 0.14 (400)	18 ± 0.33 (40)
**4g**	14 ± 0.23 (400)	12 ± 0.10 (400)	-	20 ± 0.34 (4)
**5a**	28 ± 0.09 (0.04)	30 ± 0.15 (0.04)	-	20 ± 0.32 (4)
**5b**	16 ± 0.32 (40)	25 ± 0.42 (0.4)	16 ± 0.12 (40)	14 ± 0.50 (400)
**5c**	14 ± 0.18 (400)	22 ± 0.24 (40)		18 ± 0.54 (40)
**5d**	22 ± 0.09 (4.0)	22 ± 0.20 (4.0)	16 ± 0.23 (40)	20 ± 0.12 (4)
**5e**	14 ± 0.00 (400)	16 ± 0.00 (40)	25 ± 0.13 (0.4)	18 ± 0.23 (40)
**5f**	12 ± 0.33 (400)	16 ± 0.36 (40)	-	16 ± 0.43 (40)
**5g**	14 ± 0.32 (400)	12 ± 0.30 (400)	14 ± 0.43 (400)	20 ± 0.21 (4)
Ciprofloxacin	26 ± 0.025 (0.4)	26 ± 0.45 (0.4)	23 ± 0.42 (4.0)	25 ± 0.44 (0.4)

MIC (μg/mL), minimum inhibitory concentration. Hyphens denote no activity. ^a^The concentration is 4.0 mg/mL. ^b^The values, including the diameter of the well (8 mm), are means of the three replicates.

The results revealed that all the tested compounds showed variable antibacterial activity against the Gram-positive as well as the Gram-negative bacteria. Among the tested compounds, the antibacterial activity of compounds **4a** and **5a** with a zone of inhibition of 28 mm (MIC 0.04 μg/mL) was found to be better than that of the reference drug ciprofloxacin with a zone of inhibition of 26 mm (MIC 0.4 μg/mL) against *B. subtilis*. Compounds **4f** and **5d** also displayed appreciable activity with a zone of inhibition of 24 mm (MIC 0.4 μg/mL) and 22 mm (MIC 4.0 μg/mL), respectively, against *B. subtilis*. Compound **5a** with two sulfonamide groups was found to be the most effective against *S. aureus*, showing a maximum zone of inhibition of 30 mm (MIC 0.04 μg/mL). **4a** also showed antibacterial activity with a zone of inhibition of 26 mm (MIC 0.4 μg/mL) comparable to the reference drug ciprofloxacin with a zone of inhibition of 26 mm (MIC 0.4 μg/mL) against *S. aureus*. Six more compounds (**4b-c**, **4f,** and **5b-d**) were found to possess appreciable antibacterial activity with a zone of inhibition greater than 20 mm against *S. aureus*. Interestingly, compounds **4a** and **5a**, both with an unsubstituted phenyl ring at C-3 of pyrazole, displayed a tenfold MIC (0.04 μg/mL) better than the standard drug ciprofloxacin against *B. subtilis*. A comparison within each series suggested that any substituent on the phenyl ring placed at the 3-position of the pyrazole moiety has a negative effect on the antibacterial activity against Gram-positive bacteria, as best results were seen with the naked phenyl ring in each series (compare **4a** with **4a-g**, and **5a** with **5a-g**; Table 1). No definite trend was discernable that could lead to draw a correlation of the activities between series **4** and **5**.

Against Gram-negative bacteria (*P*. *fluorescens*), only compound **5e** showed a significant activity with a zone of inhibition of 25 mm (MIC 0.4 μg/mL) comparable to the standard drug ciprofloxacin (zone of inhibition 23 mm), albeit with a tenfold better MIC. Against *E. coli*, five compounds (**4e, 4g 5a**, **5d**, and **5g**) showed good antibacterial activity with a zone of inhibition of 20 mm. However, none of the compounds were found to be as effective as the standard drug ciprofloxacin against *E. coli* (Table 1). Thus, it can be concluded that the synthesized compounds are more effective against the Gram-positive bacteria than the Gram-negative bacteria.

#### ***In vitro antifungal activity***

All the synthesized compounds were also evaluated for their *in vitro* antifungal activity against the two pathogenic fungal strains *Candida albicans* (MTCC 227) and *Saccharomyces cerevisiae* (MTCC 170) by agar well diffusion method (Table 2). Fluconazole was used as the reference drug. Most of the tested compounds in each series (**4** and **5**) showed moderate to good antifungal activity. Compound **4g** was found to be as effective as the standard drug, with a zone of inhibition of 16 mm against *C. albicans*. Against *S. cerevisiae*, **4a** and **5b** (zone of inhibition 28 mm) were found to be the most effective and were better than the standard drug fluconazole (zone of inhibition 24 mm), while the three other compounds (**4d**, **5e**, and **5g**) were found to possess good antifungal activity. Interestingly, some of the newly synthesized compounds (**4a**, **5b**, **5e**, and **5g**) showed multifold reduction in the MIC values against *S. cerevisiae*, making the new derivatives attractive agents for further evaluation.

**Table 2:** ***In vitro *antifungal activity and MIC of compounds 4 and 5 using agar well diffusion method**

**Compound**^**a**^	**Diameter of zone of inhibition in mm (MIC)**^**b**^	
	***Candida albicans***	***Saccharomyces cerevisiae***
**4a**	12 ± 0.43 (400)	28 ± 0.15 (0.04)
**4b**	12 ± 0.56 (400)	16 ± 0.38 (40)
**4c**	12 ± 0.54 (400)	16 ± 0.40 (40)
**4d**	14 ± 0.14 (400)	20 ± 0.45 (4.0)
**4e**	12 ± 0.45 (400)	18 ± 0.16 (40)
**4f**	12 ± 0.32 (400)	16 ± 0.27 (40)
**4g**	16 ± 0.27 (40)	18 ± 0.48 (40)
**5a**	12 ± 0.32 (400)	16 ± 0.00 (40)
**5b**	12 ± 0.33 (400)	28 ± 0.28 (0.04)
**5c**	-	16 ± 0.00 (40)
**5d**	12 ± 0.23 (400)	18 ± 0.18 (40)
**5e**	13 ± 0.37 (400)	22 ± 0.44 (4.0)
**5f**	-	16 ± 0.30 (40)
**5g**	12 ± 0.43 (400)	20 ± 0.10 (4.0)
Fluconazole	16 ± 0.45 (40)	24 ± 0.50 (40)

MIC (μg/mL), minimum inhibitory concentration. Hyphen denotes no activity. ^a^The concentration is 4.0 mg/mL. ^b^The values, including the diameter of the well (8 mm), are means of the three replicates.

## Experimental

The melting points were determined in open capillaries in an electrical apparatus and were uncorrected. The IR spectra in KBr were recorded with the ABB MB3000 DTGS IR instrument. The ^1^H NMR spectra were recorded on Bruker instrument (Bruker Scientific Instruments, MA, USA) at 300 MHz, taking DMSO-*d*_6_ as the solvent. The chemical shifts are expressed in *δ*, ppm. The mass spectra (DART-MS) were recorded on a JEOL AccuTOF JMS-T100LC mass spectrometer having a direct analysis in real time (DART) source in the ES^+^ mode. The purity of the compounds was checked using ^1^H NMR and thin layer chromatography on silica gel plates, using a mixture of petroleum ether and ethyl acetate as the eluent. Iodine or UV lamp was used as a visualizing agent. In the following section, these abbreviations are used: ‘s’ for singlet, ‘m’ for multiplet, and ‘ex’ for exchangeable proton are used for the NMR assignments; ‘s’ for strong and ‘m’ for medium are used for the IR assignments.

### General procedure for the conversion of 4-formylyrazole into 4-arylidene pyrazole derivatives (4 and 5)

To a solution of 4-formylpyrazoles, compound **2** (1 mmol) in ethanol was added to the appropriate pyrazolone **3** (1 mmol) followed by a catalytic amount of triethylamine and refluxed the resulting reaction mixture for 6 to 7 h. After the completion of the reaction, the solution was reduced to 1/4 of its volume and cooled to room temperature. The solid separated out was filtered, washed with water (100 mL) followed by cold ethanol (10 mL), and crystallized from ethanol to afford the target compounds **4** and **5**.

#### ***4-{4-[(3-methyl-5-oxo-1-phenyl-1,5-dihydro-4H-pyrazol-4-ylidene)methyl]-3-phenyl-1H-pyrazol-1-yl}benzenesulfonamide (4a)***

M.p. 272°C to 275°C, yield 73%; IR (KBr, cm^-1^): 3,356, 3,267, and 3,256 (m, N-H stretch), 1,674 (s, C=O stretch), 1,589 (s, C=N stretch), 1,497 (s, N-H bend), 1,311 and 1,157 (s, SO_2_ stretch); ^1^H NMR (300 MHz, DMSO-*d*_*6*_): *δ* 10.23 (s, 1H, C=CH), 8.13 (d, 2H, *J* = 7.5 Hz, Ar-H), 8.04 (d, 2H, *J* = 8.4 Hz, Ar-H), 7.95 (d, 2H, *J* = 8.4 Hz, Ar-H), 7.77 to 7.80 (m, 2H, Ar-H), 7.60 to 7.62 (m, 3H, pyrazole C_5_-H, Ar-H), 7.52 (s, ex, 2H, SO_2_NH_2_), 7.46 (t, 2H, *J* = 7.5 Hz, Ar-H), 7.21 to 7.23 (m, 1H, Ar-H), 2.23 (s, 3H, CH_3_); DART MS: *m/z* 484.30 [M + H]^+^, C_26_H_21_N_5_O_3_SH^+^ Calcd. 484.13.

#### ***4-{4-[(3-methyl-5-oxo-1-phenyl-1,5-dihydro-4H-pyrazol-4-ylidene)methyl]-3-(4-methylphenyl)-1H-pyrazol-1-yl}benzenesulfonamide (4b)***

M.p. 158°C to 160°C, yield 74%; IR (KBr, cm^-1^): 3,742, 3,272, and 3,126 (m, N-H stretch), 1,682 (s, C=O stretch), 1,589 (s, C=N stretch), 1,497 (s, N-H bend), 1,319 and 1,149 (s, SO_2_ stretch); ^1^H NMR (300 MHz, DMSO-*d*_*6*_): *δ* 10.23 (s, 1H, C=CH), 8.14 (d, 2H, *J* = 8.7 Hz, Ar-H), 8.04 (d, 2H, *J* = 8.7 Hz, Ar-H), 7.95 (d, 2H, *J* = 7.8 Hz, Ar-H), 7.68 (d, 2H, *J* = 7.8 Hz, Ar-H), 7.53 to 7.54 (m, 3H, pyrazole C_5_-H, SO_2_NH_2_), 7.47 (d, 2H, *J* = 7.8 Hz, Ar-H), 7.43 (d, 2H, *J* = 7.5 Hz, Ar-H), 7.21 to 7.24 (m, 1H, Ar-H), 2.43 (s, 3H, CH_3_), 2.25 (s, 3H, CH_3_); DART MS: *m/z* 498.30 [M + H]^+^, C_27_H_23_N_5_O_3_SH^+^ Calcd. 498.15.

#### ***4-{3-(4-methoxyphenyl)-4-[(3-methyl-5-oxo-1-phenyl-1,5-dihydro-4H-pyrazol-4-ylidene)methyl]-1H-pyrazol-1-yl}benzenesulfonamide (4c)***

M.p. 156°C to 158°C, yield 76%; IR (KBr, cm^-1^): 3,372, 3,271, and 3,123 (m, N-H stretch), 1,674 (s, C=O stretch), 1,597 (s, C=N stretch), 1,504 (s, N-H bend), 1,319 and 1,157 (s, SO_2_ stretch); ^1^H NMR (300 MHz, DMSO-*d*_*6*_): *δ* 10.24 (s, 1H, C=CH), 8.15 (d, 2H, *J* = 8.1 Hz, Ar-H), 8.04 (d, 2H, *J* = 7.8 Hz, Ar-H), 7.97 (d, 2H, *J* = 7.5 Hz, Ar-H), 7.74 (d, 2H, *J* = 7.8 Hz, Ar-H), 7.55 (s, 1H, pyrazole C_5_-H), 7.52 (s, ex, 2H, SO_2_NH_2_), 7.48 (d, 2H, *J* = 8.1 Hz, Ar-H), 7.16 to 7.22 (m, 3H, Ar-H), 3.87 (s, 3H, OCH_3_), 2.28 (s, 3H, CH_3_); DART MS: *m/z* 514.29 [M + H]^+^, C_27_H_23_N_5_O_4_SH^+^ Calcd. 514.15.

#### ***4-{4-[(3-methyl-5-oxo-1-phenyl-1,5-dihydro-4H-pyrazol-4-ylidene)methyl]-3-(4-fluorophenyl)-1H-pyrazol-1-yl}benzenesulfonamide (4d)***

M.p. 258°C to 260°C, yield 74%; IR (KBr, cm^-1^): 3,333, 3,225, and 3,132 (m, N-H stretch), 1,674 (s, C=O stretch), 1,597 (s, C=N stretch), 1,504 (s, N-H bend), 1,311 and 1,157 (s, SO_2_ stretch); ^1^H NMR (300 MHz, DMSO-*d*_*6*_): *δ* 10.25 (s, 1H, C=CH), 8.16 (d, 2H, *J* = 8.4 Hz, Ar-H), 8.05 (d, 2H, *J* = 8.7 Hz, Ar-H), 7.96 (d, 2H, *J* = 8.4 Hz, Ar-H), 7.84 to 7.89 (m, 2H, Ar-H), 7.53 (br s, 3H, pyrazole C_5_-H, SO_2_NH_2_), 7.44 to 7.46 (m, 4H, Ar-H), 7.20 to 7.24 (m, 1H, Ar-H), 2.28 (s, 3H, CH_3_); DART MS: *m/z* 502.30 [M + H]^+^, C_26_H_20_FN_5_O_3_SH^+^ Calcd. 502.13.

#### ***4-{3-(4-chlorophenyl)-4-[(3-methyl-5-oxo-1-phenyl-1,5-dihydro-4H-pyrazol-4-ylidene)methyl]-1H-pyrazol-1-yl}benzenesulfonamide (4e)***

M.p. 236°C to 238°C, yield 74%; IR (KBr, cm^-1^): 3,372, 3,271, and 3,123 (m, N-H stretch), 1,674 (s, C=O stretch), 1,589 (s, C=N stretch), 1,497 (s, N-H bend), 1,319 and 1,157 (s, SO_2_ stretch); ^1^H NMR (300 MHz, DMSO-*d*_*6*_): *δ* 10.23 (s, 1H, C=CH), 8.14 (d, 2H, *J* = 8.7 Hz, Ar-H), 8.05 (d, 2H, *J* = 8.7 Hz, Ar-H), 7.95 (d, 2H, *J* = 8.1 Hz, Ar-H), 7.83 (d, 2H, *J* = 8.4 Hz, Ar-H), 7.68 (d, 2H, *J* = 8.4 Hz, Ar-H), 7.53 (s, ex, 2H, SO_2_NH_2_), 7.51 (s, 1H, pyrazole C_5_-H), 7.43 (d, 2H, *J* = 7.8 Hz Ar-H), 7.21 to 7.24 (m, 1H, Ar-H), 2.27 (s, 3H, CH_3_); DART MS: *m/z* 518.26/520.25 [M + H]^+^/[M + H + 2]^+^, C_26_H_20_ClN_5_O_3_SH^+^ Calcd. 518.10/520.10.

#### ***4-{3-(4-bromophenyl)-4-[(3-methyl-5-oxo-1-phenyl-1,5-dihydro-4H-pyrazol-4-ylidene)methyl]-1H-pyrazol-1-yl}benzenesulfonamide (4f)***

M.p. 162°C to 165°C, yield 70%; IR (KBr, cm^-1^): 3,373, 3,261, and 3,123 (m, N-H stretch), 1,682 (s, C=O stretch), 1,597 (s, C=N stretch), 1,497 (s, N-H bend), 1,311 and 1,157 (s, SO_2_ stretch); ^1^H NMR (300 MHz, DMSO-*d*_*6*_): *δ* 10.20 (s, 1H, C=CH), 8.11 (d, 2H, *J* = 8.7 Hz, Ar-H), 8.04 (d, 2H, *J* = 8.7 Hz, Ar-H), 7.92 to 7.99 (m, 3H, Ar-H), 7.80 (d, 2H, *J* = 8.1 Hz, Ar-H), 7.67 to 7.74 (m, 3H, Ar-H), 7.61 (s, 1H, pyrazole C_5_-H), 7.54 (s, ex, 2H, SO_2_NH_2_), 7.20 to 7.26 (m, 1H, Ar-H), 2.24 (s, 3H, CH_3_); DART MS: *m/z* 562.20/564.19 [M + H]^+^/[M + H + 2]^+^, C_26_H_20_BrN_5_O_3_SH^+^ Calcd. 562.05/564.05.

#### ***4-{4-[(3-methyl-5-oxo-1-phenyl-1,5-dihydro-4H-pyrazol-4-ylidene)methyl]-3-(4-nitrophenyl)-1H-pyrazol-1-yl}benzenesulfonamide (4g)***

M.p. 310°C to 312°C, yield 73%; IR (KBr, cm^-1^): 3,371, 3,271, and 3,124 (m, N-H stretch), 1,674 (s, C=O stretch), 1,589 (s, C=N stretch), 1,497 (s, N-H bend), 1,335 and 1,157 (s, SO_2_ stretch); ^1^H NMR (300 MHz, DMSO-*d*_*6*_): *δ* 10.23 (s, 1H, C=CH), 8.44 (d, 2H, *J* = 8.4 Hz, Ar-H), 8.16 (d, 2H, *J* = 8.4 Hz, Ar-H), 8.04 to 8.11 (m, 4H, Ar-H), 7.94 (d, 2H, *J* = 7.8 Hz, Ar-H), 7.56 (s, 1H, pyrazole C_5_-H), 7.54 (s, ex, 2H, SO_2_NH_2_), 7.43 to 7.48 (m, 2H, Ar-H), 7.21 to 7.30 (m, 1H, Ar-H), 2.29 (s, 3H, CH_3_); DART MS: *m/z* 529.28 [M + H]^+^, C_26_H_20_N_6_O_5_SH^+^ Calcd. 529.12.

#### ***4-[4-({1-[4-(aminosulfonyl)phenyl]-3-methyl-5-oxo-1,5-dihydro-4H-pyrazol-4-ylidene}methyl)-3-phenyl-1H-pyrazol-1-yl]benzenesulfonamide (5a)***

M.p. 248°C to 252°C, yield 73%; IR (KBr, cm^-1^): 3,340, 3,271, and 3,217 (m, N-H stretch), 1,674 (s, C=O stretch), 1,589 (s, C=N stretch), 1,504 (s, N-H bend), 1,335 and 1165 (s, SO_2_ stretch); ^1^H NMR (300 MHz, DMSO-*d*_*6*_): *δ* 10.21 (s, 1H, C=CH), 8.14 to 8.19 (m, 4H, Ar-H), 8.05 (d, 2H, *J* = 8.4 Hz, Ar-H), 7.91 (d, 2H, *J* = 8.7 Hz, Ar-H), 7.79 to 7.80 (m, 2H, Ar-H), 7.59 to 7.63 (m, 4H, pyrazole C_5_-H, Ar-H), 7.54 (s, ex, 2H, SO_2_NH_2_), 7.36 (s, ex, 2H, SO_2_NH_2_), 2.28 (s, 3H, CH_3_); DART MS: *m/z* 563.28 [M + H]^+^, C_26_H_22_N_6_O_5_S_2_H^+^ Calcd. 563.10.

#### ***[4-({1-[4-(aminosulfonyl)phenyl]-3-methyl-5-oxo-1,5-dihydro-4H-pyrazol-4-ylidene}methyl)-3-(4-methylphenyl)-1H-pyrazol-1-yl]benzenesulfonamide (5b)***

M.p. 282°C to 285°C, yield 75%; IR (KBr, cm^-1^): 3,340, 3,248 (m, N-H stretch), 1,682 (s, C=O stretch), 1,589 (s, C=N stretch), 1,497 (s, N-H bend), 1,304 and 1,149 (s, SO_2_ stretch);^1^H NMR (300 MHz, DMSO-*d*_*6*_): *δ* 10.19 (s, 1H, C=CH), 8.13 to 8.19 (m, 4H, Ar-H), 8.05 (d, 2H, *J* = 8.4 Hz, Ar-H), 7.92 (d, 2H, *J* = 8.4 Hz, Ar-H), 7.68 (d, 2H, *J* = 7.8 Hz, Ar-H), 7.58 (s, 1H, pyrazole C_5_-H), 7.52 (s, ex, 2H, SO_2_NH_2_), 7.42 (d, 2H, *J* = 7.5 Hz, Ar-H), 7.34 (s, ex, 2H, SO_2_NH_2_), 2.43 (s, 3H, CH_3_), 2.28 (s, 3H, CH_3_); DART MS: *m/z* 577.32 [M + H]^+^, C_27_H_24_N_6_O_5_S_2_H^+^ Calcd. 577.12.

#### ***4-[4-({1-[4-(aminosulfonyl)phenyl]-3-methyl-5-oxo-1,5-dihydro-4H-pyrazol-4-ylidene}methyl)-3-(4-methoxyphenyl)-1H-pyrazol-1-yl]benzenesulfonamide (5c)***

M.p. 320°C to 326°C, yield 74%; IR (KBr, cm^-1^): 3,317, 3,232, 3,148 and 3,086 (m, N-H stretch), 1,674 (s, C=O stretch), 1,589 (s, C=N stretch), 1,504 (s, N-H bend), 1,335 and 1157 (s, SO_2_ stretch); ^1^H NMR (300 MHz, DMSO-*d*_*6*_): *δ* 10.18 (s, 1H, C=CH), 8.12 to 8.19 (m, 4H, Ar-H), 8.04 (d, 2H, *J* = 8.7 Hz, Ar-H), 7.91 (d, 2H, *J* = 8.7 Hz, Ar-H), 7.73 (d, 2H, *J* = 8.7 Hz, Ar-H), 7.57 (s, 1H, pyrazole C_5_-H), 7.53 (s, ex, 2H, SO_2_NH_2_), 7.36 (s, ex, 2H, SO_2_NH_2_), 7.16 (d, 2H, *J* = 8.4 Hz, Ar-H), 3.86 (s, 3H, OCH_3_), 2.28 (s, 3H, CH_3_); DART MS: *m/z* 593.30 [M + H]^+^, C_27_H_24_N_6_O_6_S_2_H^+^ Calcd. 593.12.

#### ***4-[4-({1-[4-(aminosulfonyl)phenyl]-3-methyl-5-oxo-1,5-dihydro-4H-pyrazol-4-ylidene}methyl)-3-(4-fluorophenyl)-1H-pyrazol-1-yl]benzenesulfonamide (5d)***

M.p. 208°C to 210°C, yield 71%; IR (KBr, cm^-1^): 3,317, 3,202, 3,148 and 3,070 (m, N-H stretch), 1,674 (s, C=O stretch), 1,589 (s, C=N stretch), 1,504 (s, N-H bend), 1,335 and 1,157 (s, SO_2_ stretch); ^1^H NMR (300 MHz, DMSO-*d*_*6*_): *δ* 10.20 (s, 1H, C=CH), 8.14 to 8.19 (m, 4H, Ar-H), 8.05 (d, 2H, *J* = 8.7 Hz, Ar-H), 7.91 (d, 2H, *J* = 8.7 Hz, Ar-H), 7.86 (dd, 2H, *J* = 8.1, 5.4 Hz Ar-H), 7.55 (br s, 3H, pyrazole C_5_-H, SO_2_NH_2_), 7.43 to 7.49 (m, 2H, Ar-H), 7.37 (s, ex, 2H, SO_2_NH_2_), 2.29 (s, 3H, CH_3_); DART MS: *m/z* 581.26 [M + H]^+^, C_26_H_21_FN_6_O_5_S_2_H^+^ Calcd. 581.10

#### ***4-[4-({1-[4-(aminosulfonyl)phenyl]-3-methyl-5-oxo-1,5-dihydro-4H-pyrazol-4-ylidene}methyl)-3-(4-chlorophenyl)-1H-pyrazol-1-yl]benzenesulfonamide (5e)***

M.p. 318°C to 320°C, yield 72%; IR (KBr, cm^-1^): 3,340, 3,256, 3,148 and 3,086 (m, N-H stretch), 1,682 (s, C=O stretch), 1,597 (s, C=N stretch), 1,497 (s, N-H bend), 1,335 and 1,157 (s, SO_2_ stretch); ^1^H NMR (300 MHz, DMSO-*d*_*6*_): *δ* 10.20 (s, 1H, C=CH), 8.15 to 8.19 (m, 4H, Ar-H), 8.06 (d, 2H, *J* = 8.4 Hz, Ar-H), 7.92 (d, 2H, *J* = 8.7 Hz, Ar-H), 7.84 (d, 2H, *J* = 8.1 Hz, Ar-H), 7.68 (d, 2H, *J* = 8.1 Hz, Ar-H), 7.58 (s, 1H, pyrazole C_5_-H), 7.54 (s, ex, 2H, SO_2_NH_2_), 7.36 (s, ex, 2H, SO_2_NH_2_), 2.31 (s, 3H, CH_3_); DART MS: *m/z* 597.25/599.25 [M + H]^+^/[M + H + 2]^+^, C_26_H_21_ClN_6_O_5_S_2_H^+^ Calcd. 597.07/599.07.

#### ***4-[4-({1-[4-(aminosulfonyl)phenyl]-3-methyl-5-oxo-1,5-dihydro-4H-pyrazol-4-ylidene}methyl)-3-(4-bromophenyl)-1H-pyrazol-1-yl]benzenesulfonamide (5f)***

M.p. 330°C to 332°C, yield 69%; IR (KBr, cm^-1^): 3,742, 3,333 and 3,256 (m, N-H stretch), 1,682 (s, C=O stretch), 1,597 (s, C=N stretch), 1,497 (s, N-H bend), 1,335 and 1,157 (s, SO_2_ stretch); ^1^H NMR (300 MHz, DMSO-*d*_*6*_): *δ* 10.19 (s, 1H, C=CH), 8.15 to 8.19 (m, 4H, Ar-H), 8.06 (d, 2H, *J* = 8.4 Hz, Ar-H), 7.92 (d, 2H, *J* = 8.4 Hz, Ar-H), 7.82 (d, 2H, *J* = 7.8 Hz, Ar-H), 7.76 (d, 2H, *J* = 7.8 Hz, Ar-H), 7.57 (s, 1H, pyrazole C_5_-H), 7.54 (s, ex, 2H, SO_2_NH_2_), 7.35 (s, ex, 2H, SO_2_NH_2_), 2.31 (s, 3H, CH_3_); DART MS: *m/z* 641.19/643.20 [M + H]^+^/[M + H + 2]^+^, C_26_H_21_BrN_6_O_5_S_2_H^+^ Calcd. 641.02/643.02.

#### ***4-[4-({1-[4-(aminosulfonyl)phenyl]-3-methyl-5-oxo-1,5-dihydro-4H-pyrazol-4-ylidene}methyl)-3-(4-nitrophenyl)-1H-pyrazol-1-yl]benzenesulfonamide (5g)***

M.p. 298°C to 300°C, yield 71%; IR (KBr, cm^-1^): 3,348, 3,248, 3,132 and 3,109 (m, N-H stretch), 1,682 (s, C=O stretch), 1,589 (s, C=N stretch), 1,528 (s, N-H bend), 1,335 and 1,157 (s, SO_2_ stretch); ^1^H NMR (300 MHz, DMSO-*d*_*6*_): *δ* 10.21 (s, 1H, C=CH), 8.45 (d, 2H, *J* = 8.4 Hz, Ar-H), 8.16 to 8.19 (m, 4H, Ar-H), 8.11 (d, 2H, *J* = 9.0 Hz, Ar-H), 8.07 (d, 2H, *J* = 8.7 Hz, Ar-H), 7.92 (d, 2H, *J* = 8.7 Hz, Ar-H), 7.62 (s, 1H, pyrazole C_5_-H), 7.55 (s, ex, 2H, SO_2_NH_2_), 7.36 (s, ex, 2H, SO_2_NH_2_), 2.33 (s, 3H, CH_3_); DART MS: *m/z* 608.27 [M + H]^+^, C_26_H_21_N_7_O_7_S_2_H^+^ Calcd. 608.09.

## Conclusions

In conclusion, we have presented the novel 4-arylidene pyrazole derivatives bearing benzenesulfonamide moiety as potential antimicrobial agents. The reported compounds were conveniently prepared by Knovenagel condensation of 4-formyl pyrazoles with pyrazolones. Some of the tested compounds displayed excellent antibacterial properties against Gram-positive bacteria (*B. subtilis* and *S. aureus*). For instance, compounds **4a** and **5a** were found to be more effective than the reference drug ciprofloxacin. However, against Gram-negative bacteria (*P. fluorescens* and *E. coli*), the level of activity shown by the tested compounds was found to be significantly low as only 2 of the 14 tested compounds displayed activities comparable to the reference drug against *P. fluorescens*, while none of the compounds were found to be highly effective against *E. coli.* All the tested compounds showed moderate antifungal activity against *C. albicans*, while two compounds showed activity better than the reference drug against *S. cerevisiae*. In short, the reported compounds showed remarkable potential as antimicrobial agents and warranted further investigation of their mechanism of actions and binding site. The studies regarding these aspects are being planned with the help of a prospective collaborator.

## Abbreviations

DMSO: Dimethylsulfoxide; MIC: Minimum inhibitory concentration; MTCC: Microbial-type culture collection.

## Competing interests

The authors declare that they have no competing interests.
